# Editorial: Power, discrimination, and privilege in individuals and institutions

**DOI:** 10.3389/fpsyg.2024.1376169

**Published:** 2024-03-19

**Authors:** Sonya C. Faber, Monnica T. Williams, Matthew D. Skinta

**Affiliations:** ^1^School of Psychology, University of Ottawa, Ottawa, ON, Canada; ^2^Roosevelt University, Chicago, IL, United States

**Keywords:** power dynamics, systemic discrimination, racism, institutional bias, mental health, policy

“The system is not much concerned if any individual swaps places between levels. The system is concerned that the edifice itself remains intact.”—*Marie Laurencin*

## Introduction

We are very pleased to introduce this Research Topic in *Frontiers* on the topic of “*Power, discrimination, and privilege in individuals and institutions*”. People and systems they create are rife with prejudices, leading to discrimination and inequitable outcomes. Problems operating in oppressive systems include racism, casteism, colorism, sexism, heterocentrism, ethnocentrism, and their intersections. These biases cause issues such as rejection of stigmatized groups, structural racism, disenfranchisement of women, barriers to higher education, economic oppression, radicalization, and colonialism. In this Research Topic, we take a closer look to find the core of the problem, which is inevitably an imbalance in the distribution of power and its misuse.

This Research Topic contains 20 articles that cover a range of critical issues in Psychology (Personality and Social, Forensic and Legal, Cultural, and Gender, Sex and Sexualities) and Sociology (Race and Ethnicity, and Gender, Sex and Sexualities). These articles originate with researchers from countries including Germany, China, Singapore, Romania, the USA, and Canada. The researchers submitting these articles identify with a range of ethnicities, including Roma, Indigenous Australian, African American, Mexican American, Southeast Asian, Jewish Canadian, and Black German to name a few.

## Systems of injustice

**Figure 1:**
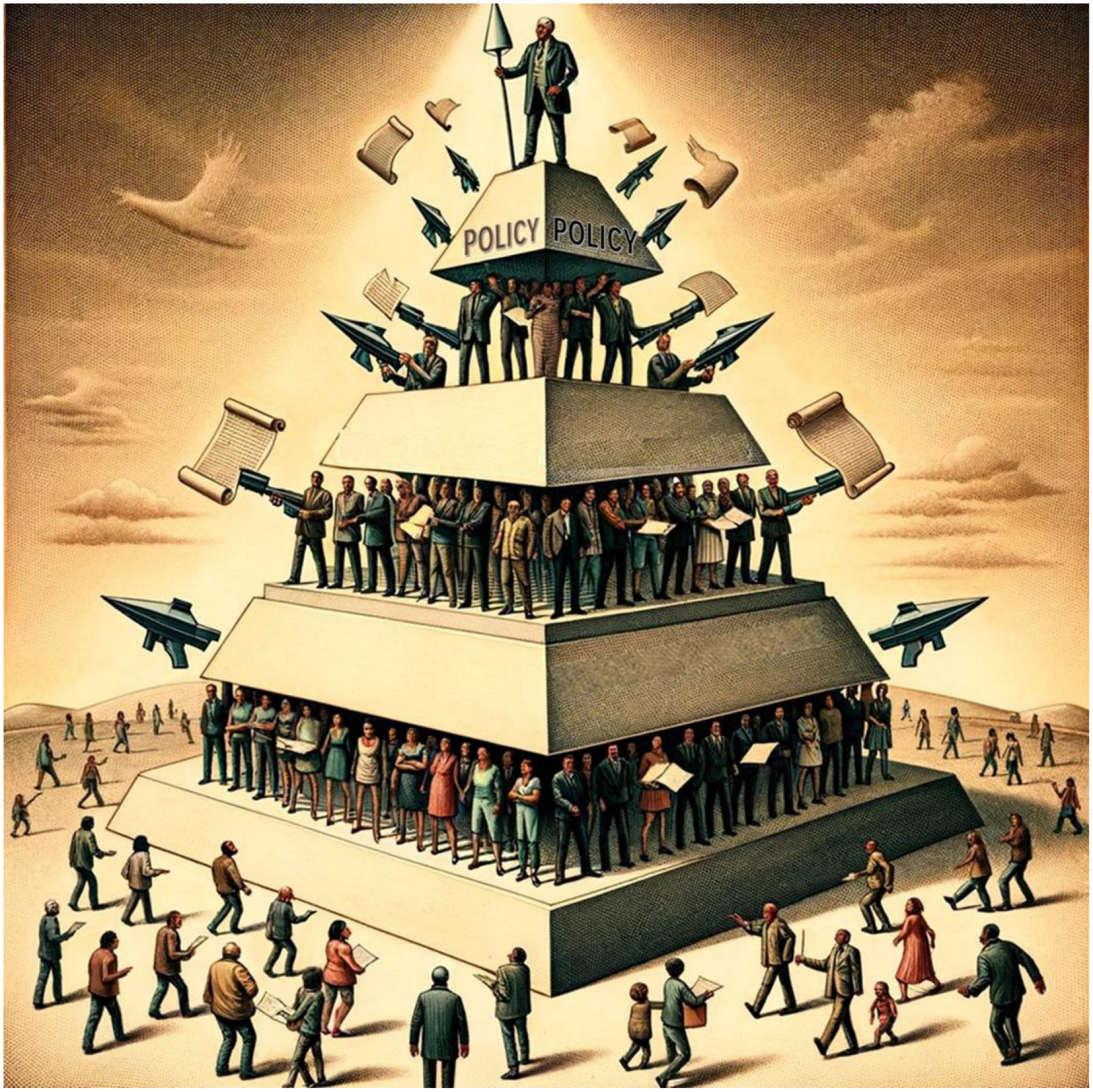
Depiction of Weaponization of Policy. Policies are the framework for the system which becomes invisible for those who live within it, unable to perceive the ingrained injustices, and well-defended, making it difficult to change. Image generated by OpenAI's DALL-E.

Systems of injustice can be found anywhere power is concentrated, including board rooms, editorial offices, university admissions policies, legislative bodies, and organizational bylaws. Policies and procedures may seem fair and appropriate on their face, but, in their use, end up bolstering systems that support hierarchical, non-meritocratic outcomes. The name for this is called *weaponization of policy* (Figure 1). This type of problem was evident in a paper by Faber, Wu et al. in our Research Topic that uncovered the abuse of power in the disciplinary actions of a state psychology licensing board, where inequitable outcomes were particularly devastating for disempowered early career psychologists. Since the release of those findings, the corresponding state psychological organization decided to poll its members on their experiences with the licensing board, and the problems pointed out by Faber, Wu et al. were supported by survey results (KPA, 2023), hopefully leading to reform.

Likewise, our professional societies, and by extension the products produced by them, are also rife with biases. Faber, Metzger et al. took a deep dive into an organization with a stated vision of “alleviat[ing] human suffering,” the Association for Contextual and Behavioral Science (ACBS), to uncover anti-Black racism in their membership, organizational procedures, and scholarship that emerged from their journals (e.g., Misra et al., 2023). The outcome of this investigation was documenting that no Black people had held leadership positions in the organization at the time that article was submitted, severe unaddressed experiences of racism by Black ACBT members, and sparse scholarship about clinical uses for ACT for that demographic.

These systems of injustice are also at work in our legal systems. Kantachote (2024) describes the struggle of Thai American massage businesses, which are subject to heightened surveillance and regulation due to stereotypical assumptions based on race, gender, and ethnicity. These inequities result in hardships that threaten the livelihood of hard-working business owners and their staff. Black American men face this same sort of intersectional oppression. Smith and Harris advance the notion of “bad faith” as the connecting thread that permits unfreedom and inequities despite cherished US ideals of freedom and equality. This paper highlights the unique issues of race and masculinity in the oppression of Black men, past, and present.

These problems are not just found in the United States but are worldwide. Goghari and Kusi offer a comprehensive and compelling introduction to the key elements of the caste system of India, underscoring the many factors that maintain this problematic categorization of people, despite strong policies enacted that aim to remedy historic and current wrongs that lead to social disadvantage.

Yuen et al. examine barriers to the pursuit of higher education among multicultural youth in Hong Kong. They found that Chinese immigrant and ethnic minority South Asian youth often encounter a glass ceiling and financial aid barriers to pursuing higher education, in contrast to their Hong Kong mainstream counterparts.

Likewise, Davidson et al. found gender, ethnicity, and nationality to be key issues causing stress among postdoctoral researchers in German academia. These early career researchers are subject to tenuous working conditions that pose significant challenges to the pursuit of a long-term research career, particularly for international scientists and those from marginalized groups.

## Personal prejudice

There is a synergistic connection between institutional and individual prejudice, as they serve to maintain one another. Pascal et al. present new research on the topic of emotional relevance and prejudice, where they test the effect of disgust on prejudice toward two different stigmatized European ethnic groups. One of these groups is the Roma people, who experience severe discrimination and exclusion, contributing to their impoverished status. Pascal et al. showed that priming subjects with images to elicit feelings of disgust make them feel more unfavorably toward Roma people, who are often stereotyped as being unhygienic. This suggests that presenting stigmatized groups in stereotypical ways potentiates stereotypes, and by extension, perhaps presenting these groups in counter-stereotypical ways can help reduce prejudice.

## Mental health consequences

When larger systems and powerful individuals are free to engage in bias against outgroups it can lead to various forms of oppression, resulting in negative psychological effects among those who are disempowered. Holmes et al. present the findings from the development of a new clinical measure called the Oppression-Based Traumatic Stress Inventory (OBTSI). Validated on a student and outpatient sample, this measure offers a novel approach to measuring the impact of intersectional discriminatory trauma using DSM-5 PTSD criteria. Having multiple stigmatized identities correlates with greater oppression-based trauma (Williams et al., 2023b). As such, tools like these are an important means of helping clinicians and researchers quantify the suffering caused by marginalization due to such identities.

Sometimes, the pain of being stigmatized causes the oppressed to lash out against the very society that caused them pain. Shafieioun and Haq cogently explain the process of radicalization from a societal perspective in their qualitative study of how people become extremists. Using interviews with ex-militants of the radical group, Islamic State of Iraq and Syria (ISIS), they show how problems such as social injustice, misuse of power, marginalization, and discrimination, can serve as key factors leading some individuals to identify and sympathize with radical ideology.

Mental health clinicians have their own ways of managing the impact of racialization within their practice, and this differs based on racial identity. In their qualitative study of therapists, Bergkamp et al. describe how the way in which clinical psychologists are taught to provide patients with care can actually harm both patients and psychologists because of its lack of attention to the role race plays in psychological health and traumatization. The authors investigate the impact of socially-conferred privilege, particularly in race and gender dynamics, on the therapeutic relationship and professionalization process in psychotherapy. Their findings reveal distinct experiences for BIPOC vs. white psychologists. Neither are adequately prepared to provide care when confronted with clients of different racial backgrounds suggesting an urgent need for comprehensive changes in training models, continuing education, and supervision to address the psychological impact of race and privilege in the field. In regards to racial dynamics, this paper cogently points out how psychologists need to heal themselves before they can heal others.

## Bringing light into covert systems for change

It is critical to expose covert, invisible, or under-examined aspects of power to shine a light on behaviors that bolster misuse of power by individuals and institutions in order to know where to affect change. Many of those who hold and wield power do not recognize or acknowledge that they are exercising power at all. It is important to be able to define, measure and call-out hidden power while also advancing mechanisms to support healing and harmony. Faber and Williams tackle this issue in higher education in their paper about racial and gender dynamics in university classrooms. They describe how toxic femininity is used by White women to control or derail conversations about race at the expense of students of color. They also describe strategies professors can use to recenter the conversation on people of color and maintain order in the classroom.

Morisano et al. address the issues around the conduct of research with Indigenous peoples in Canada, with a focus on ethical and policy considerations. The mental health and wellness of Indigenous Peoples is compromised by policies that ignore Indigenous rights, that frame colonization as historical rather than ongoing, or that minimize the impact of assimilation. The authors call for autonomous control over research involving Indigenous People, and ultimately a much needed research paradigm shift.

Vierra et al. wrote about critical action to redress systemic oppression using a person-centered approach. This study used mixed methods to understand why some choose to participate in more impactful forms of activism, such as Black Lives Matter, while others opt for superficial or performative action to advocate for people who experience systemic oppression. Those who were most active in their approach to racial justice had a greater critical consciousness, whereas those who were passive tended to endorse racial colorblindness. Racial colorblindness is a form of racism whereby advocates prefer not to consider or discuss racial differences (Kanter et al., 2017).

## Reclaiming personal power

Experiencing any form of discrimination can leave targets feeling disempowered and demoralized. Huang offers insights about power and self-esteem by highlighting the moderating effect of self-defense mechanisms. This study explores the dynamics of personal power in social relationships, finding that loss of power does not necessarily result in a decline in self-esteem; rather, personal self-defense mechanisms play a moderating role, indicating that constant power maintains or increases self-esteem, especially when self-defense levels are higher. Notably, when faced with social downfall, people in power are more prone to find ways to deceive themselves rather than learn humility.

Barrita and Wong-Padoongpatt conducted a study of ethnic identity and resilience, to better understand protective factors against self-blame in the face of racial microaggressions. This US based cross-sectional study involving 696 diverse participants revealed that self-blame mediates the relationship between racial microaggressions and psychological distress. Embracing one's cultural identity, along with being mentally strong in tough situations, can help reduce the emotional impact of racial microaggressions, but this seems to work best for those who strongly identify with their ethnicity and have high mental resilience. Maintaining a strong ethnic identity therefore offers some protection against the negative effects of microaggressions, as we have seen in studies of African Americans (Williams et al., 2012), but does not eliminate the negative emotional impact.

Traversa et al. examine the widely maligned construct of “cancel culture”, and find it can actually be useful insomuch that it provides collective validation for groups experiencing harm against the background of a majority culture that historically dismisses or invalidates the perceptions of marginalized group members when it differs from the perspectives of majority group members. This novel research addresses the impact of cancel culture on marginalized groups, finding that episodes of cancel culture can positively influence collective action intentions by fostering feelings of collective validation. This sheds light on the dynamics involved in resistance strategies of marginalized communities and the importance of an external environment that reflects the experiences of marginalized individuals.

## Compassion and connection

Dismantling systems of oppression require the efforts of people in both privileged and marginalized groups to effect change. Allies are members of dominant groups that work to uplift and create equity for disempowered groups. Empathy and compassion have been implicated as important prerequisites for social justice allyship and antiracism work (Gonzalez et al., 2015; Williams et al., 2021). Karnaze et al. underscore how racist systems impact the wellbeing of minoritized individuals, emphasizing the potential for accelerated systemic reforms through greater ally support. Their research found that allyship was correlated with both empathy and compassion, but empathy was the stronger predictor. This contrasts with a trend in recent literature to consider compassion as the more reliable driver of behavioral change compared with empathy (e.g., Lim and DeSteno, 2016). They provide insights for future research and interventions to dismantle structural racism focused on exercises that focus on connecting with the feelings of racialized people through sharing personal narratives.

The Louisiana Contextual Science Research Group explored the role of vulnerability in intimacy, emphasizing that safe and functional intimacy emerges when vulnerability is consensual, empowered, and positively reinforced—with the responsibility for promoting this dynamic resting on the individual with more power in the interaction. In short, fostering healthy intimacy involves consensual and empowered vulnerability, with the person in a position of influence playing a critical role in creating a safe space for such interactions. This reminds us that those who are in positions of power bear the brunt of the responsibility for creating safe spaces for people in marginalized groups.

## The struggle is real

One reason we sought to create this Research Topic is because we found there were few academic havens for scholarship focused on the misuse of power and systems of oppression. Strauss et al., members of our own research team, described the problem of racism and censorship in the editorial and peer review process, and we learned that even in the course of curating our own Research Topic, we would need to navigate this challenge at every turn. This was evident at each stage, from submissions we never saw because they were filtered out too early in the editorial process (deemed a poor fit), to peer-reviewers who changed their mind about reviewing papers once they realized the topic was about bias in their cherished systems, to author positionality statements that bizarrely vanished between the proofing and publications stage. These problems are not unique to *Frontiers*, and indeed the very fact we were able to create this Research Topic is a testament to the dedication of the publishers to make this Research Topic happen. These barriers typically prevent scholarship that calls out institutional problems from ever seeing the light of day. Yet, despite our many successes, there were several worthy papers that ended up being rejected by forces beyond our reach. We were astonished to learn that a paper could be rejected even after passing editorial and peer-review. This reminds us that bias and oppression are still present, even in our own systems, and much more is needed to completely dismantle them.

## Conclusion

There is an inverse relationship between power hoarding (avoidance of risk) and courage (acceptance of risk) and their effects on personal moral growth (Williams et al., 2023a). This also applies to systems of power particularly in the construction and administration of institutional policy. Policy is a power tool that can result in outcomes that advantage an ingroup but seem to be fair on the surface, and can maintain plausible deniability by allowing exceptions. Such policy is designed to covertly support biased systems and is the institutionalized correlate of individual aversive racism. One hoped for outcome in this Research Topic was a larger showing of scholarship focused on institutional systems of inequity. Perhaps unsurprisingly, in hindsight, we found that this type of manuscript was the most difficult to shepherd through the publication process. This includes a manuscript that called out problems in mental health care policy and another that underscored problematic racial dynamics of an organization's board of directors. These powerful systems are self-protecting, and as such, finding ways to bring light to these issues will require thoughtful intervention at many levels.

## Author contributions

SF: Conceptualization, Writing – review & editing. MW: Conceptualization, Writing – original draft, Writing – review & editing. MS: Writing – review & editing.
